# Functional Evaluation of the Visual Pathway in Patients with Multiple Sclerosis Using a Multifunction Stimulator Monitor

**DOI:** 10.1155/2019/2890193

**Published:** 2019-09-18

**Authors:** M. Satue, L. Jarauta, J. Obis, M. Cipres, M. J. Rodrigo, C. Almarcegui, I. Dolz, J. R. Ara, J. Martin, L. E. Pablo, E. Garcia-Martin

**Affiliations:** ^1^Ophthalmology Department, Miguel Servet University Hospital, Zaragoza, Spain; ^2^IIS-Aragon, Aragon Health Research Institute, Zaragoza, Spain; ^3^Neurology Department, Miguel Servet University Hospital, Zaragoza, Spain; ^4^Neurophysiology Department, Miguel Servet University Hospital, Zaragoza, Spain

## Abstract

**Objectives:**

To assess the capability of the vision monitor unit Monpack One of detecting visual function alterations in patients with multiple sclerosis (MS) and to evaluate the correlation between structural retinal parameters and functional measurements obtained with this device.

**Methods:**

Forty-eight patients with MS and 46 healthy controls were included in a cross-sectional study. All participants underwent a complete functional evaluation of the visual pathway, which included low-contrast visual acuity (LCVA), contrast sensitivity vision (CSV), automated perimetry, multifocal visual evoked potentials (mfVEPs), and pattern electroretinogram (ERG). All tests were performed using the vision monitor unit Monpack One (Metrovision, France), a multifunction stimulator device. Retinal structural measurements were obtained in all subjects using Triton swept source optical coherence tomography (Topcon, Japan).

**Results:**

Patients with MS presented reduced low-contrast VA (*p* < 0.001) and reduced CSV at medium (*p*=0.001, *p*=0.013) and low (*p*=0.001, *p*=0.002) spatial frequencies. All visual field parameters were found to be altered in MS patients compared with controls (≤0.001). Patients with MS presented lower amplitude of the P100 waveform of the mfVEP in areas corresponding to central (*p* < 0.001), inferonasal (*p*=0.001), and inferotemporal (*p*=0.003) retina. The pattern ERG did not show significant differences. Significant correlations were observed between structural retinal measurements and functional parameters, especially between the inner macular areas and measurements corresponding to contrast sensitivity and perimetry indexes.

**Conclusions:**

Patients with MS present visual dysfunction detectable with the vision monitor unit Monpack One. This device may be a fast and useful tool to provide a full evaluation of axonal damage in patients with multiple sclerosis.

## 1. Introduction

Multiple sclerosis (MS) is a multifocal central nervous system disorder characterized by inflammatory demyelinating lesions affecting white and gray matter. Even without a history of optic neuritis (ON), optic nerve atrophy and thinning of the peripapillary retinal nerve fiber layer are two typical findings of patients suffering from MS [1].

Axonal loss is considered to be the main cause of progressive disability in MS [1–3], and neuronal loss is increasingly recognized as a biomarker that correlates with disability in these patients [4–7]. MS is often associated with involvement of the visual pathway that can lead to clinically evident manifestations (such as ON and diplopia) and more frequently, to subclinical alterations. Decreased contrast sensitivity and color vision deficiencies have been widely observed in MS [8–10] and have been correlated with poorer performances in everyday tasks, such as driving and reading.

Previous research on functional evaluation of the visual pathway demonstrated altered responses in MS. Visual evoked potentials (VEPs) and pattern electroretinogram (ERG) show frequent abnormalities in these patients [11–13]. Asymptomatic visual field disturbances seem to be present also in MS patients without a previous episode of optic neuritis [14]. Recent studies have correlated alterations in functional responses with structural changes in the retina of MS patients [10, 11].

In this study, we evaluated the visual pathway of MS patients using the vision monitor unit Monpack One, a multifunction stimulator device which integrates different functional, electrophysiological, and psychophysical tests for the complete evaluation of visual function parameters. The main objective of this study was to assess the capability of this device of detecting visual function abnormalities through different tests (visual acuity, contrast sensitivity vision, perimetry, visual evoked potentials, and pattern electroretinogram) in patients with MS and to analyze the correlation between the functional measurements obtained and retinal structural parameters measured with swept source optical coherence tomography (SS-OCT).

## 2. Methods

Patients with definite relapsing-remitting (RR) MS were included in this observational cross-sectional study. A total of 48 eyes of 48 patients and 46 eyes of 46 healthy individuals were evaluated. All procedures adhered to the tenets of the Declaration of Helsinki, and all participants provided written informed consent to participate in the study.

The diagnosis of MS was based on the McDonald criteria and confirmed by a neurologist [15]. Related medical records were carefully evaluated, and information about Expanded Disability Status Scale (EDSS) scores, disease duration and subtype, and modifying disease treatments and prior episodes of ON was recorded. Only patients with RR MS were included in our study. Patients with a visual acuity <0.1 (using Snellen scale), intraocular pressure >20 mmHg, refractive errors >5 diopters (D) of equivalent spherical or 3 D of astigmatism, active MS outbreaks (of any neurologic deficit), and/or history of a previous episode of ON were excluded from our study. The reason to exclude patients with a prior ON episode was that visual function loss secondary to ON is widely demonstrated, but the main purpose of this study is to check if neuronal damage secondary to MS itself (i.e., chronic neurodegeneration) causes subclinical visual affectation. The diagnosis of ON was based on reports from the patient and the treating neurologist and clinical findings such as decrease in visual acuity, visual defects in the perimetry, color vision loss, relative afferent pupillary defect, and papillary pallor observed in the funduscopy.

All eyes underwent a complete neuro-ophthalmic evaluation that included pupillary, anterior segment, and funduscopic examination to detect any ocular pathology that might affect visual function tests.

Visual function was evaluated with the vision monitor unit Monpack One (Metrovision, France), a multifunction stimulator device which integrates different functional, electrophysiological, phychophysical, and oculomotor tests for a complete evaluation of visual function parameters. The vision monitor comprises a central liquid crystal display panel with LED backlight and four peripheral panels illuminated with LEDs. A light sensor placed anteriorly provides feedback to the entire system allowing a constant luminance during the test performance. In our study, visual acuity (VA), contrast sensitivity vision (CSV), visual field, multifocal PEV, and pattern ERG were registered and analyzed in all eyes.

Low-contrast VA was evaluated at a contrast level of 10%. The percentage indicates the level of contrast; that is, 100% would represent black letters over white background, and 10% represents medium gray letters over white background. Two different ETDRS charts were selected (one chart for each eye) to avoid learning and memory bias. Measurements were obtained under scotopic light conditions, at a distance of 4 meters in monocular vision using best correction. Visual results were recorded and registered as a VA result (expressed in logMar) as well as the number of total read letters.

Static CSV was evaluated using a sinusoidal pattern (grid) at different spatial frequencies: 0.5, 1, 2, 5, 10, and 20 cycles per degree. Threshold CSV was analyzed by progressive increase of 0.25 dB of contrast before the test performance. For contrast sensitivity evaluation, the patient/control pressed a push-button when the contrast grid was first visible in each spatial frequency. Measurements were obtained at 2 meters of distance under scotopic light conditions and monocular vision. CSV results were registered as a sensitive curve in dB (0 representing no contrast sensitivity).

Visual field was assessed using the vision monitor Fast Perimetry 30 protocol (Fast-30), which analyzes 94 points over the central visual field. The test was performed in monocular vision using near distance correction. Fixation was controlled through a central video camera. Quality parameters (i.e., fixation and attention) were analyzed, and tests with poor performances (>5/15 fixation losses and >5/15 attention losses) were automatically rejected by the program. Results were presented as a 2D and 3D sensitivity map (in dB), a color probability score map, and a sensitivity curve. The average deficit (AD), corrected average deficit (CAD), variance of deficits (VD), and temporal fluctuation (TF) were recorded.

Electrophysiological evaluation included multifocal VEPs and pattern ERG. Multifocal VEPs were assessed using alternate checkboards with a central fixation point. Electrical responses were recorded using four electrodes attached to the subject scalp by subcutaneous needles (inion: 4 cm above the inion, 1 cm above the inion, and 4 cm right/left of the inion). The ground electrode was placed in the subject's vertex. VEPs were evaluated with the MVEP35 procedure, which analyzes 35 zones for each eye. The test was performed at a 33 cm distance, under scotopic conditions after 2 minutes light adaptation, using near distance correction in monocular vision. Fixation was controlled through a central video camera. Results were presented as analytic data, amplitude histogram, color map, and 3D representation for 5 different retinal areas: central, superonasal, superotemporal, inferonasal, and inferotemporal. Amplitude of P100 wave for each retinal area was recorded and expressed in nV/deg^2^.

The pattern ERG was evaluated using alternate checkboards with a central fixation point. Electrical responses were recorded using a total of 5 DTL (Dawson–Trick–Litzkow) electrodes: 2 electrodes (one for the right eye and one for the left eye) draped horizontally across the cornea at the level of the lower lid (prior topical anesthesia) and 2 reference electrodes positioned at the outer canthus (right and left eye). The ground electrode was placed at Fpz. The pattern ERG uses a reversal stimulation pattern covering a field of 60 degrees with large patterns for 50 minutes. The test was performed at a distance of 30 cm, under scotopic conditions after 2 minutes of light adaptation, using near distance correction in binocular vision. Results are expressed as a graphic of the electrical response registered and as the analytic data of the different spikes selected. Amplitude and latency of the N35, P50, and N95 waves (spikes) were recorded.

Structural measurements of the retina were obtained using the DRI Triton SS-OCT device (Topcon, Tokyo, Japan) in all eyes. The 3D wide protocol was used, which includes a wide scanning range that focuses on both the macular (ETDRS: Early Treatment Diabetic Retinopathy Study) and the peripapillary (TSNIT: temporal-superior-nasal-inferior-temporal) area. With the ETDRS scan, full retinal thickness in nine macular areas (which include a central 1 mm circle representing the fovea, and inner and outer rings measuring 3 mm and 6 mm in diameter, respectively) was analyzed; the TSNIT scan provides automated separate measurements of different retinal layers: retinal nerve fiber layer (RNFL), ganglion cell layer (GCL), retinal thickness, and the choroidal plexus. The TSNIT provides measurements of the 4 peripapillary quadrants (superior, nasal, inferior, and temporal), 6 sectors (superonasal, superotemporal, nasal, temporal, inferonasal, and inferotemporal), and 12 clock sectors. Only measurements of the GCL in the 6 peripapillary sectors were evaluated in this study. All scans were obtained by the same experienced operator and were checked by an experienced rater for quality of the segmentation immediately after acquisition. The DRI Triton SS-OCT provides a quality scale in the image to indicate the signal strength. The quality score ranges from 0 (poor quality) to 100 (excellent quality). Only images with a score >55 were analyzed in our study; poor quality images were rejected and recaptured prior to data analysis.

All data analyses were performed using SPSS software version 20.0 (SPSS Inc., Chicago, IL). The Kolmogorov–Smirnov test was used to assess sample distribution. Differences between evaluations of MS patients and healthy subjects were compared using the Mann–Whitney *U* test, as the sample did not correspond to a normal distribution (Kolmogorov test: *p* < 0.05). Correlation between functional and structural data of all subjects (patients and controls) was assessed using Spearman's rho test. Correlation between the EDSS score and visual function parameters was also analyzed. Each eye was considered separately, and only one eye from each patient was randomly selected for analysis.

## 3. Results

Forty-eight patients with MS and 46 healthy controls were included in the study. The mean age of the patients with MS was 49.25 (SD = 12.98), and the mean age of the healthy controls was 45.74 (SD = 10.52). The female/male ratio was 4/1 in both groups. Age, sex, and intraocular pressure did not differ significantly between healthy controls and patients with MS (*p*=0.154, 0.145, and 0.770, respectively).

### 3.1. Visual Function Analysis

All patients had been diagnosed with MS relapse-remitting subtype and were under treatment with interferon (50%), glatiramer acetate (12.5%), or fingolimod (8.3%). Only 29.2% of the patients were not under any current treatment. The mean EDSS score was 2.03 (SD = 0.54).

Patients with MS presented reduced low-contrast VA (0.43 ± 0.50 in patients vs. 0.08 ± 0.27 in controls, *p* < 0.001) and reduced number of read letters (31.90 ± 8.20 letters vs. 39.70 ± 5.58 letters, *p* < 0.001) compared with healthy controls (see Table 1).

CSV was found to be reduced in MS patients in all spatial frequencies. CSV at medium frequencies (2 cpd: 21.29 ± 2.95 in patients vs. 22.06 ± 2.12 in controls, *p*=0.001; 5 cpd: 20.34 ± 1.68 vs. 21.91 ± 1.94, *p*=0.013) and low frequencies (1 cpd: 19.09 ± 1.55 vs. 20.50 ± 2.02, *p*=0.001; 0.5 cpd: 15.98 ± 1.69 vs. 17.04 ± 2.03, *p*=0.002) was significantly reduced in patients compared with controls (see Table 1).

All visual field parameters were found to be altered in MS patients compared with controls. The AD (*p* < 0.001), the CAD (*p* < 0.001), the VD (*p* < 0.001), and the TF (*p*=0.001) were significantly reduced in patients compared with healthy subjects (see Table 1).

Patients with MS presented lower amplitude of the P100 waveform in the multifocal VEP. Significant differences between patients and controls were observed in the areas corresponding to central (798.80 ± 585.58 nV/deg^2^ in patients vs. 1556.81 ± 1120.97 nV/deg^2^ in controls, *p* < 0.001), inferonasal (523.90 ± 262.71 nV/deg^2^ vs. 798.50 ± 390.14 nV/deg^2^, *p*=0.001), and inferotemporal (677.55 ± 730.19 nV/deg^2^ vs. 830.40 ± 380.09 nV/deg^2^, *p*=0.003) retina (see Table 2). Patients with MS also presented delayed latency in the N35, P50, and N95 waveforms of the pattern ERG. However, significant differences between patients and controls were not found in any of the pattern ERG responses (see Table 2).

**Figure 1:**
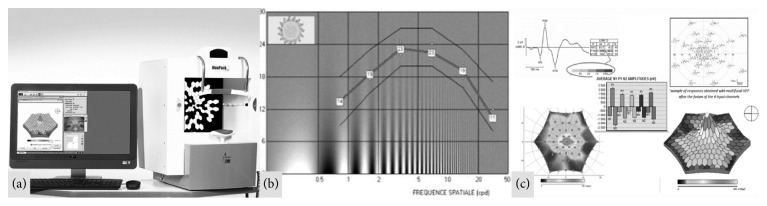
(a) Monpack One monitor. (b) Displayed report of the contrast sensitivity vision test in one of our controls. (c) Displayed report of multifocal visual evoked potentials in one of our controls.

Reports from CSV and mfVEPs as provided by Monpack One are seen in Figure 1.

### 3.2. Structural Evaluation

Patients presented significantly reduced macular retinal thickness (*p* < 0.001) in all evaluated ETDRS areas and reduced peripapillary GCL thickness (*p* < 0.001) in all 6 sectors analyzed, compared to healthy controls (see Supplementary Table [Supplementary-material supplementary-material-1]).

### 3.3. Correlation Analysis

A significant correlation was observed between macular parameters and functional measurements obtained with the Monpack One device. The inner superior and inner inferior areas correlated most strongly with contrast sensitivity parameters (the strongest correlation was observed between inner superior and 5 cpd, *r* = 0.36, *p* < 0.001). All inner sectors of the macular ETDRS ring presented a positive correlation with the number of read letters (superior: *r* = 0.37, *p* < 0.001; nasal: *r* = 0.21, *p*=0.042; inferior: *r* = 0.27, *p*=0.009; and temporal: *r* = 0.26, *p*=0.011) and an inverse association with all visual field parameters (the strongest correlation was observed between inner superior and VD, *r* = 0.35, *p*=0.001). Central thickness correlated with the P100 amplitude of central retina measured with the mfVEP (*r* = 0.31, *p*=0.002). No correlations were found between functional parameters and the outer macular areas. Results are shown in Tables 3 and 4.

Peripapillary GCL thickness (total and the temporal quadrant) correlated significantly with most functional parameters analyzed with the Monpack One device. A positive correlation was observed between total GCL thickness and CSV at 1, 2, 5, and 10 cpd (the strongest correlation at 10 cpd, *r* = 0.28, *p*=0.007), and a negative association was observed between total GCL thickness and visual field parameters (the strongest correlation with CAD, *r* = −0.32, *p*=0.002). A positive correlation between total GCL and the VEP in central retina (*r* = 0.26, *p*=0.012) was observed. Temporal GCL thickness correlated with CSV at 2, 5, 10, and 20 cpd (the strongest at 10 cpd, *r* = 0.40, *p* < 0.001) and all visual field parameters (the strongest association with CAD, *r* = −0.36, *p* > 0.001). Additionally, a significant relationship was observed between GCL thickness and letter read (total GCL: *r* = 0.35, *p*=0.001, and temporal GCL: *r* = 0.39, *p* < 0.001). Results are shown in Table 3.

No significant correlations were observed between ERG and structural parameters.

The amplitude of mfVEPs in the central retina was inversely correlated with the EDSS score (*r* = −0.443, *p*=0.002). No other significant associations between functional parameters and the EDSS score were observed.

## 4. Discussion

In the present study, we evaluated visual function parameters of 48 MS patients and 46 healthy controls using the vision monitor unit Monpack One, a multiple function stimulator device which integrates different functional, electrophysiological, and phychophysical tests for a complete evaluation of visual function. To the best of our knowledge, this is the first study featuring visual function tests with Monpack One in patients with a neurodegenerative disease. Our patients presented lower VA, decreased CSV affecting medium and lower frequencies, increased defects in the visual field, and reduced amplitude in the P100 component of the multifocal VEP affecting the central and inferior sectors of the retina. All these altered parameters were observed using one single device, the vision monitor unit Monpack One.

Visual dysfunction was previously reported in MS patients and may occur in up to 80% of cases during the course of the disease [16]. Contrast sensitivity provides more complete information about visual function than visual acuity tests. Measures of low-contrast vision and CSV were sensitive to visual impairment, even in patients with VA of 20/20 or better (measured with a Snellen chart), and have been correlated with poorer performances in everyday tasks [8, 17–20]. Our patients presented reduced CSV in low and midspatial frequencies and low-contrast VA compared with healthy controls, which is consistent to previous research involving classic visual function tests [11, 21, 22] and tests performed with other video processors [23, 24]. However, high spatial frequencies were not significantly affected in our patients, contrary to previous findings [11, 24]. More studies including a larger sample size would be needed to establish whether the Monpack One device detects significant differences in CSV at high frequencies in MS.

Patients suffering from MS present visual field defects compared with healthy controls, according to previous published research [25, 26]. However, other studies suggested different results [27, 28]. Despite the fact that visual field defects are more frequently reported in patients with MS and previous history of ON [29], asymptomatic visual field disturbances seem to be present also in patients without a previous episode of ON [14].

Our patients presented significant alterations in all visual field parameters compared with healthy controls, which are consistent with results provided by Pueyo et al. and Castro et al. Since none of our patients had a previous history of ON, our results also agree with results provided by Chorazy et al. and strengthen the evidence of visual field abnormalities in non-ON MS patients.

Electrophysiological responses of the visual pathway are also altered in patients with MS [11, 30, 31]. According to previous published studies, MS patients present increased latency and decreased amplitude in the P100 waveform of the pattern VEP [11, 27, 32, 33]. The pattern VEP provides information for a complete study of the visual pathway, from the optic nerve to the visual cortex. The pattern VEP has been shown to be more sensitive than perimetry, contrast sensitivity, and retinal structural defects at detecting hidden visual loss in patients with MS with 20/20 vision and without history of optic neuritis [30, 34]. However, the pattern VEP is dominated by macular responses, thus other focal defects may not be detected. The multifocal VEP captures a significantly larger area of the visual field than the pattern VEP and can provide information of the global optical path, topographic assessment of amplitude and latency, and thus detects focal and peripheral defects [35]. Recent studies have shown the sensitivity of the technique in identifying multifocal VEP defects after recovery from an episode of ON [36] and also in patients suffering from MS (with and without history of ON) [37]. Our patients presented decreased amplitude of P100 waveform in the multifocal VEP (which represents neurodegeneration), especially those corresponding to central, inferonasal, and inferotemporal retina. Focal defects are not usually reported when performing pattern VEPs [11].

The pattern ERG is considered to be the most useful neurophysiologic technology for detecting abnormalities in the visual pathway caused by neurodegenerative diseases [38]. Although the exact origin of the pattern ERG is not clear, it seems to be generated by the inner layers of the central retina (ganglion cells and their axons) [11], so it reflects a more specific involvement of the retinal nerve fiber layer anomalies than the alterations detected by PEV. Previous research demonstrated increased latency of the N95 waveform of the pattern ERG in patients with MS and an increased P50/N95 ratio [11]. In the present study, MS patients presented increased latencies of the N95 waveform which is consistent with previous reports. However, this difference between patients and controls was not found to be significant, probably due to the small sample size of our study.

Structural changes in the retina of patients with MS have been widely studied. Optic nerve atrophy and thinning of the peripapillary retinal nerve fiber layer are two typical findings of patients with MS. Hitherto, studies using spectral-domain OCT have revealed that the retina in non-ON eyes shows peripapillary thinning compared to healthy controls [39, 40]. The most recent milestone in the development of retina and choroid visualization strategies is swept source OCT (SS-OCT), with a scan speed of 100,000 A-scans/sec, which provides more accurate three-dimensional images of the retina and the choroid than previous spectral-domain devices [41]. In our study, we used Triton SS-OCT to evaluate the retina of patients with MS. Furthermore, we analyzed the correlation between functional measurements obtained with the Monpack One monitor and structural data measured with Triton OCT. The correlation between functional changes in patients with MS [10] and other neurodegenerative diseases [42] and retinal structural measurements has been previously evaluated using spectral-domain OCT devices. To the best of our knowledge, this is the first study assessing the correlation between visual function parameters obtained with a multifunction simulator device and SS-OCT technology. Our analysis revealed that contrast sensitivity parameters and visual field data correlated most significantly with macular and peripapillary measurements. However, the strength of the association was limited. Correlations between functional and structural retinal parameters are seldom strong or perfect, since changes in structural parameters are not happening at the same time as functional changes. This is frequently observed in glaucoma and also in neurodegenerative diseases such as MS, and Parkinson electrophysiological tests performed with the Monpack One device demonstrated barely no association with obtained structural data, opposite to previous studies where significant association between structural changes and electroretinogram/multifocal visual evoked potentials was observed [43, 44]. Nonparametric tests (as the one used in our calculations) tests are more demanding to accept statistical differences; this also might explain why we did not find more association between parameters. Additionally, devices used in previous studies (to obtain both structural and functional data) differ from what was used in our present study; we believe results might not be entirely comparable. However, our results suggest that the correlation between functional data obtained with the multifunction simulator device and structural retinal measurements may only be applicable to CSV tests and perimetry data. In addition, there were no observable differences between MS patients and controls in the ERG measurements (opposite to previous reported data). Taken altogether, these results might suggest that the ability of the ERG function of the Monpack One monitor should be improved. However, more studies using the multifunction simulator device Monpack One are needed to corroborate our findings.

The main goal of this study was to analyze the capability of the vision monitor unit Monpack One of detecting visual dysfunction in MS. Low-contrast VA, CSV, visual field parameters, and multifocal VEPs were significantly altered in our patients, and these results agree with previous published research. Additionally, since none of our patients presented any previous episode of optic neuritis, which causes inflammation and retrograde retinal cells alteration, we are certain that results in our patients were associated with disease itself and not with previous inflammatory episodes of the optic nerve. Moreover, similar alterations in visual function tests in these patients have been associated with axonal loss secondary to MS [10, 11].

In conclusion, patients with MS and without ON antecedent present visual dysfunction detectable with the vision monitor unit Monpack One. Measurements obtained with this device correlate with structural retinal data obtained with SS-OCT technology. Monpack One may be a fast and useful tool to provide a complete evaluation of axonal damage in patients with MS, although some of the electrophysiological tests might require further improvement.

## Figures and Tables

**Table 1:** Visual acuity, contrast sensitivity vision, and visual field parameters (standard deviation) in patients with multiple sclerosis and healthy controls, as measured with the Monpack One device.

Functional parameters	MS patients	Controls	*p*
*Contrast vision*			
VA ETDRS 10%	0.43 (0.50)	0.08 (0.27)	**<0.001**
Read letters	31.90 (8.20)	39.70 (5.58)	**<0.001**
CSV 0.5 cpd	15.98 (1.69)	17.04 (2.03)	**0.002**
CSV 1 cpd	19.09 (1.55)	20.50 (2.02)	**0.001**
CSV 2 cpd	21.29 (2.95)	22.06 (2.12)	**0.001**
CSV 5 cpd	20.34 (1.68)	21.91 (1.94)	**0.013**
CSV 10 cpd	19.35 (2.60)	21.83 (2.84)	0.088
CSV 20 cpd	11.73 (4.31)	15.17 (3.21)	0.101

*Visual field parameters*			
Avg. deficit	0.14 (0.35)	1.64 (2.87)	**<0.001**
Corrected avg. deficit	0.20 (0.46)	1.57 (2.49)	**<0.001**
Variance of deficits	4.05 (6.03)	14.65 (16.52)	**<0.001**
Temporal fluctuation	0.98 (0.45)	1.73 (1.34)	**<0.001**

Bold letters indicate statistical significance (*p* < 0.05). Abbreviations: MS, multiple sclerosis; VA, visual acuity; CSV, contrast sensitivity vision; cpd, cycles per degree; avg., average.

**Table 2:** Electrophysiological parameters (standard deviation) in patients with multiple sclerosis and healthy controls, as measured with the Monpack One device.

Electrophysiological parameters	MS patients	Controls	*p*
*mfVEPs (nV/deg* ^*2*^)			
Central	798.80 (585.58)	1556.81 (1120.9)	**<0.001**
Superonasal	504.00 (278.84)	637.95 (675.843)	0.455
Superotemporal	533.31 (292.29)	579.58 (341.27)	0.610
Inferonasal	523.90 (262.71)	798.50 (390.14)	**0.001**
Inferotemporal	677.55 (730.19)	830.40 (380.09)	**0.003**

*Pattern ERG*			
N35 latency (msec)	27.18 (29.31)	23.82 (5.34)	0.515
N35 amplitude (mV)	−0.70 (1.34)	−0.62 (2.45)	0.534
P50 latency (msec)	46.59 (10.05)	43.34 (15.17)	0.471
P50 amplitude (mV)	4.43 (4.79)	7.21 (11.32)	0.097
N95 latency (msec)	83.09 (21.06)	71.07 (25.97)	0.070
N95 amplitude (mV)	−3.27 (5.38)	−4.79 (6.30)	0.184

Bold letters indicate statistical significance (*p* < 0.05). Abbreviations: MS, multiple sclerosis; mfVEPs, multifocal visual evoked potentials; ERG, electroretinogram.

**Table 3:** Correlation between visual function parameters obtained with the Monpack one device and structural parameters (macular and peripapillary) obtained with Triton optical coherence tomography.

Visual function parameters	Inner superior macula		Inner inferior macula		Total GCL thickness		Temporal GCL thickness	
	*r*	*p*	*r*	*p*	*r*	*p*	*r*	*p*
VA 10%	0.17	0.96	0.78	0.455	0.18	0.079	0.26	**0.010**
Letter read	0.37	**<0.001**	0.27	**0.009**	0.35	**0.001**	0.39	**<0.001**
CSV 0.5	0.18	0.083	0.09	0.370	0.15	0.131	0.20	0.053
CSV 1	0.27	**0.007**	0.18	0.069	0.23	**0.023**	0.20	**0.048**
CSV 2	0.34	**0.001**	0.25	**0.013**	0.23	**0.024**	0.25	**0.016**
CSV 5	0.36	**<0.001**	0.25	**0.016**	0.27	**0.008**	0.33	**0.001**
CSV 10	0.33	**0.001**	0.30	**0.003**	0.28	**0.007**	0.39	**<0.001**
CSV 20	0.25	**0.014**	0.26	**0.011**	0.13	0.135	0.25	**0.013**
Avg. deficit	−0.27	**0.008**	−0.22	**0.034**	−0.27	**0.008**	−0.34	**0.001**
Corrected avg. deficit	−0.30	**0.003**	−0.26	**0.011**	−0.32	**0.002**	−0.36	**<0.001**
Variance of deficits	−0.35	**0.001**	−0.29	**0.004**	−0.25	**0.017**	−0.28	**0.005**
Temporal fluctuation	−0.30	**0.003**	−0.24	**0.019**	−o.26	**0.011**	−0.27	**0.008**

Bold letters indicate a significant correlation. Abbreviations: VA, visual acuity; CSV, contrast sensitivity vision; avg., average; GCL, ganglion cell layer.

**Table 4:** Correlation between visual field parameters obtained with the Monpack One device and macular measurements obtained with Triton optical coherence tomography.

Visual function parameters	Central		Inner superior		Inner nasal		Inner inferior		Inner temporal	
	*r*	*p*	*r*	*p*	*r*	*p*	*r*	*p*	*r*	*p*
Avg. deficit	−0.28	**0.007**	−0.27	**0.008**	−0.23	**0.025**	−0.22	**0.034**	−0.32	**0.002**
Corrected avg. deficit	−0.25	**0.016**	−0.30	**0.003**	−0.23	**0.024**	−0.26	**0.011**	−0.30	**0.003**
Variance of deficits	−0.25	**0.017**	−0.35	**0.001**	−0.25	**0.014**	−0.29	**0.004**	−0.33	**0.001**
Temporal fluctuation	−0.13	0.194	−0.30	**0.003**	−0.22	**0.028**	−0.24	**0.019**	−0.26	**0.010**

Bold letters indicate a significance <0.05. Abbreviations: avg., average.

## Data Availability

The data used to support the findings of this study are available from the corresponding author upon request.

## Supplementary Materials

Supplementary Materials Supplementary Table 1: macular and peripapillary measurements (standard deviation between parenthesis) obtained with Triton swept source optical coherence tomography in patients with multiple sclerosis and healthy controls. Bold letters indicate statistical significance (*p* < 0.05). Abbreviations: MS, multiple sclerosis.
