# Supplementary material for: Functional Evaluation of the Visual Pathway in Patients with Multiple Sclerosis Using a Multifunction Stimulator Monitor
Source: J Ophthalmol. 2019 Sep 18;2019:2890193. doi: 10.1155/2019/2890193 (PMC6769350; doi:10.1155/2019/2890193)
Supplement: Supplementary Materials — Supplementary Table 1: macular and peripapillary measurements (standard deviation between parenthesis) obtained with Triton swept source optical coherence tomography in patients with multiple sclerosis and healthy controls. Bold letters indicate statistical significance (p < 0.05). Abbreviations: MS, multiple sclerosis. [file 2890193.f1.pdf]

Structural parameter	MS	Controls	p
Macular thickness			
Central	203.42 (44.01)	222.30 (21.38)	**0.010**
Inner superior	303.46 (30.32)	324.08 (8.91)	**<0.001**
Inner nasal	308.16 (19.32)	325.12 (11.71)	**<0.001**
Inner inferior	304.51 (18.37)	319.99 (8.84)	**<0.001**
Inner temporal	292.22 (20.41)	308.80 (11.33)	**<0.001**
Outer superior	265.67 (12.46)	273.89 (11.10)	**0.001**
Outer nasal	279.21 (16.78)	290.73 (11.99)	**<0.001**
Outer inferior	254.79 (17.86)	261.93 (13.65)	**0.033**
Outer temporal	249.38 (14.25)	256.45 (15.39)	**0.023**
Average	270.54 (14.20)	281.47 (11.04)	**<0.001**
Volume	7.65 (0.40)	7.96 (0.31)	**<0.001**
Ganglion cell thickness			
Total	41.55 (3.91)	46.98 (1.90)	**<0.001**
Superotemporal	38.86 (6.36)	44.35 (6.63)	**<0.001**
Temporal	49.95 (6.90)	57.57 (4.03)	**<0.001**
Inferotemporal	38.94 (7.83)	45.13 (5.86)	**<0.001**
Superonasal	40.79 (5.67)	45.87 (5.27)	**<0.001**
Nasal	38.77 (4.09)	42.45 (2.96)	**<0.001**
Inferonasal	36.24 (7.38)	41.12 (4.25)	**<0.001**

Supplementary table 1: Macular and peripapillary measurements (standard deviation between parenthesis) obtained with Swept Source Triton Optical coherence tomography in patients with multiple sclerosis and healthy controls. Bold letters indicate statistical significance ($p<0.05$).

Abbreviations: MS, multiple sclerosis.
